# Advances and emerging technologies in the diagnosis of viral infections in pigs: Progress, challenges, and One Health perspectives

**DOI:** 10.14202/vetworld.2025.3788-3805

**Published:** 2025-12-10

**Authors:** Kydyr Nazerke, Asaubayev Ruslan, Daugaliyeva Saule, Daugaliyeva Aida, Vitmer Svetlana

**Affiliations:** 1 LLP “Scientific and Production Center, Center of Advanced Technologies in Agriculture”, 23 Karim Sutyshev, 150000, Petropavlovsk, Republic of Kazakhstan; 2 LLP “Scientific Production Center of Microbiology and Virology”, 105 Bogenbay Batyr, 050010, Almaty, Republic of Kazakhstan; 3 LLP “Kazakh Research Institute for Livestock and Fodder Production”, 51 Zhandosov, 050035, Almaty, Republic of Kazakhstan

**Keywords:** viral infections, emerging technologies, One Health, molecular diagnostics, artificial intelligence, microfluidic platforms, biosensors

## Abstract

Viral infections continue to pose major challenges to pig health, farm productivity, and global food security. Early and accurate diagnosis is the cornerstone of disease prevention, surveillance, and control in swine populations. In recent years, remarkable progress has been achieved in molecular, serological, and digital diagnostic technologies, enabling more rapid, sensitive, and field-adaptable detection of important porcine viruses such as African swine fever virus, porcine reproductive and respiratory syndrome virus, and classical swine fever virus. This review summarizes current and emerging diagnostic approaches, highlighting polymerase chain reaction (PCR) and its advanced forms, quantitative PCR and digital PCR, as the gold standards for laboratory confirmation. The advent of next-generation sequencing and metagenomics has revolutionized pathogen discovery and genomic surveillance, providing comprehensive insights into viral evolution and transboundary transmission. Isothermal amplification techniques such as loop-mediated isothermal amplification and recombinase polymerase amplification have shown strong potential for on-farm diagnosis due to their simplicity, rapidity, and minimal equipment requirements. Innovations such as clustered regularly interspaced short palindromic repeats (CRISPR)/CRISPR-associated-based assays, biosensors, lab-on-a-chip platforms, and point-of-care testing devices are bridging the gap between laboratory precision and field application, allowing rapid decision-making during outbreaks. The integration of artificial intelligence, machine learning, and geographic information systems has further enhanced diagnostic interpretation, real-time data sharing, and early outbreak prediction under the One Health framework. Despite these advances, challenges remain in ensuring assay standardization, affordability, and equitable access in resource-limited regions. Continued international collaboration, data sharing, and policy harmonization under the guidance of the Food and Agriculture Organization, the World Organization for Animal Health, and the World Health Organization are essential for the global control of swine viral diseases. Ultimately, combining molecular innovation with digital adaptability offers the most promising path toward resilient, cost-effective, and sustainable diagnostic systems for safeguarding animal and public health.

## INTRODUCTION

Pig production is a vital component of global agriculture, serving as a significant source of animal protein. However, the sector faces constant threats from viral pathogens that negatively impact animal health, productivity, and sustainability. The most important among these are African swine fever virus (ASFV), porcine reproductive and respiratory syndrome virus (PRRSV), classical swine fever virus (CSFV), porcine circovirus types 2 and 3 (PCV2 and PCV3), and swine influenza virus (SIV), all of which are highly contagious and capable of rapid transboundary spread.

Traditional diagnostic approaches, such as virus isolation, serology, and histopathology, remain important reference methods; however, they are time-consuming, require centralized laboratories, and lack sensitivity in early infection stages. For example, virus isolation in cell culture continues to be the “gold standard,” but its practical use in acute outbreaks is limited by the need for specialized facilities and long turnaround times [1, 2]. Similarly, enzyme-linked immunosorbent assay (ELISA) and other serological assays may fail to distinguish between vaccinated and naturally infected animals. Since the early 2020s, the diagnosis of swine viruses has shifted from traditional serological methods to more sensitive molecular approaches. The integration of high-throughput and point-of-care diagnostic technologies has significantly changed the detection, monitoring, and control of disease outbreaks [3]. These changes reflect technological advances and the need to adapt to the increasing complexity of the epidemiological situation and the global spread of viral infections.

The rapid evolution of viral genomes, particularly in RNA viruses such as PRRSV and SIV, further complicates diagnostics and demands frequent assay updates. Consequently, there has been a shift toward molecular and genomic approaches, including polymerase chain reaction (PCR)-based and next-generation sequencing (NGS) methods, which enable faster and more accurate pathogen detection and genotyping. The need for rapid diagnostics was particularly acute during the recent African swine fever (ASF) outbreaks in China and Eastern Europe. Delays in disease detection resulted in large-scale culling of animals and significant economic losses [1]. Veterinary laboratories worldwide have accelerated the implementation of nucleic acid-based methods, such as PCR and its modifications.

In the modern era of One Health, the health of humans, animals, and ecosystems is interconnected. Viral diseases of pigs not only impact animal production but also pose indirect risks to global biosecurity and trade stability [4, 5]. The concept of One Health emphasizes the interconnectedness of human, animal, and environmental health. Swine serve as critical intermediate hosts for several zoonotic viruses, including influenza A viruses, due to their susceptibility to both avian and human strains. This “mixing vessel” phenomenon increases the likelihood of reassortment and spillover events, posing a direct threat to public health. Modern molecular diagnostics in pigs, such as quantitative PCR (qPCR), NGS, and metagenomics, play a crucial role in identifying early genetic markers of zoonotic potential, enabling cross-sectoral response before human infection occurs.

Furthermore, NGS and artificial intelligence (AI)-assisted analytics enable the genomic surveillance (GS) of viruses such as swine influenza, hepatitis E virus, and coronaviruses to enable real-time tracking of mutations associated with host adaptation and interspecies transmission. For example, integrated bioinformatic pipelines now support the simultaneous analysis of viral genomes from animal and human samples, facilitating early warning of zoonotic outbreaks. Zoonotic influenza A viruses, particularly H1N1 and H3N2, remain critical components of swine viral surveillance because of their potential for reassortment and pandemic risk [6]. Pigs serve as “mixing vessels” for influenza viruses of avian and human origin, facilitating the emergence of novel genotypes with altered virulence or transmission potential. The 2009 H1N1 pandemic underscored the significance of influenza of swine origin in global health security.

Modern molecular assays, including multiplex reverse-transcription (RT)-qPCR and NGS, allow for precise subtyping and genomic tracking of circulating H1N1 and H3N2 variants. Integration of these data into global surveillance networks enhances early warning systems for zoonotic infections [7]. The inclusion of these subtypes in diagnostic panels supports preparedness for potential future pandemics and aligns with the One Health approach to cross-species viral monitoring.

The implementation of unified diagnostic platforms across human and veterinary laboratories enhances global preparedness for emerging viral threats in line with One Health policy frameworks advocated by the Food and Agriculture Organization (FAO), World Organization for Animal Health (WOAH, formerly OIE) and the World Health Organization. Field-deployable molecular tools (such as loop-mediated isothermal amplification [LAMP] and clustered regularly interspaced short palindromic repeats [CRISPR] assays) and AI-driven disease prediction models contribute to continuous surveillance, especially in regions with intensive pig farming. These integrated systems improve veterinary response efficiency and strengthen pandemic prevention networks by enabling timely detection, data sharing, and coordinated biosecurity measures. The globalization of livestock movement and the intensification of pig production systems have amplified the risk of the spread of transboundary diseases. Therefore, efficient and harmonized diagnostic systems are critical for ensuring early detection, containment, and effective response to emerging viral threats.

A recent surveillance study by OIE [8] has revealed the growing importance of novel enteric viruses, including rotavirus C, porcine sapelovirus, porcine kobuvirus, and porcine astrovirus, in pig populations. These viruses are increasingly recognized as the cause of neonatal diarrhea, growth retardation, and subclinical intestinal infections, which complicate coinfections with classic pathogens such as porcine epidemic diarrhea virus (PEDV) and transmissible gastroenteritis virus. Molecular detection by RT-qPCR and metagenomics has enabled the simultaneous identification of multiple enteric viruses in fecal and intestinal samples, thereby improving diagnostic accuracy. For example, kobuvirus and sapelovirus are frequently detected in mixed infections and can act as cofactors worsening enteric syndromes [9].

Advances in molecular biology, bioinformatics, and nanotechnology have dramatically reshaped the diagnostic capabilities of veterinary virology. Techniques such as LAMP and recombinase polymerase amplification (RPA) allow field-based detection of ASFV, PRRSV, and CSFV with accuracy exceeding 90%, significantly improving outbreak management [10]. NGS and metagenomics have revolutionized pathogen discovery, enabling the identification of known and unknown viral genomes within 24–72 h. These technologies support comprehensive surveillance, facilitate genomic epidemiology, and aid in understanding viral evolution and recombination events in porcine populations. However, cost, infrastructure, and bioinformatics expertise remain challenges, particularly in low- and middle-income countries where high-throughput platforms are less accessible. Thus, strengthening diagnostic networks and implementing data-sharing systems for viral genomes are essential steps toward improving diagnostic preparedness and international cooperation.

Although numerous diagnostic tools for swine viral diseases have been developed, significant limitations persist in their accessibility, affordability, and harmonization across regions. Many laboratories in developing countries lack the infrastructure to adopt advanced molecular assays such as NGS and CRISPR-based detection, resulting in delays in disease confirmation and reporting. Diagnostic assays must also be continuously updated to match viral genetic evolution, particularly in rapidly mutating RNA viruses, such as PRRSV and SIV. Furthermore, there is limited integration between veterinary and public health data systems, reducing the efficiency of One Health-based surveillance. Despite the potential of AI-assisted and portable molecular platforms, their field validation and regulatory standardization remain incomplete. Addressing these issues requires coordinated international efforts to develop standardized, cost-effective, and field-adaptable diagnostic systems capable of supporting global biosecurity and trade.

This review provides a comprehensive overview of current and emerging diagnostic technologies for major viral diseases in pigs, with a focus on molecular, serological, and metagenomic approaches. This study highlights the comparative advantages and limitations of classical and modern diagnostic tools, emphasizing point-of-care testing (POCT), CRISPR/Cas-based detection, and biosensor platforms that are transforming veterinary diagnostics [3]. Particular attention is given to integrating digital technologies, AI, and geographic information system (GIS)-based systems into swine health management, as these tools facilitate real-time data analysis and outbreak prediction. This study aims to contribute to the development of efficient, accessible, and harmonized diagnostic systems that enhance disease surveillance, control, and prevention within the global framework of sustainable animal production and One Health.

## MODERN DIAGNOSTIC APPROACHES FOR VIRAL INFECTIONS IN PIGS

The accurate detection of viral infections in pigs relies on robust laboratory methods that have undergone significant improvements over the past decade. This section reviews the key modern diagnostic approaches used worldwide, based on current scientific research and international standards.

### Importance of standardized sampling and handling

One of the key areas for further improvement in the diagnosis of viral infections in pigs is the standardization of sampling, preservation, and transportation methods. Although oral fluid has been widely used in recent years for non-invasive monitoring of infections in industrial pig farms, there are still no uniform protocols for its collection and storage.

Flores-Contreras *et al*. [3] have noted that differences in sampling device type, collection time, and storage temperature significantly affect the accuracy of ELISA and PCR diagnostics. Therefore, the implementation of standardized procedures across diagnostic laboratories is crucial to ensure consistency and reliability of results.

### Types of diagnostic specimens

Standardized protocols are critical for obtaining reliable and reproducible diagnostic results. The common clinical specimen types include serum, whole blood, oral fluid, nasal swabs, and tissue biopsies. Maintaining cold-chain conditions during transport and storage is essential to preserve sample integrity. In addition, proper labeling and documentation are vital to prevent sample degradation and cross-contamination, ensuring traceability throughout the diagnostic process.

### Environmental sampling in epidemiological surveillance

Recent studies have highlighted the potential use of environmental samples, such as air, dust, and wastewater, for the early detection of circulating viruses, including PRRSV and PCV2. These innovative approaches show promise in large-scale epidemiological surveillance; however, their effectiveness must be validated under different climatic conditions and husbandry systems to establish reliability and reproducibility.

### Overcoming logistical challenges in sample transport

The transportation of field samples remains a major bottleneck, particularly in regions with limited infrastructure or long-distance logistics. To address this issue, innovative RNA/DNA stabilizers and Flinders Technology Associates card-based carriers have been introduced. These technologies enable nucleic acid preservation at room temperature without requiring cold-chain maintenance, representing a promising advancement for resource-limited settings and field-based surveillance programs.

### Quality assurance and international standards

International Organization for Standardization/International Electrotechnical Commission

(ISO/IEC) 17025-accredited veterinary laboratories operate under strict quality assurance frameworks, ensuring high accuracy and reliability in diagnostic testing. These standards include the routine use of positive and negative controls, retesting of equivocal results, and participation in external quality assessments.

International organizations, including the WOAH and the FAO, have developed comprehensive standard operating procedures for specimen collection and laboratory diagnostics. These globally recognized guidelines promote harmonization of diagnostic approaches, facilitate data comparability, and strengthen transboundary disease surveillance networks.

## SEROLOGICAL DIAGNOSTIC METHODS FOR VIRAL INFECTIONS IN PIGS

Serological tests remain vital for diagnosing and monitoring viral infections in pigs. These tests are crucial for detecting specific antibodies or antigens in serum, providing information on past or current infections, vaccination status, and the level of population immunity.

Some of the most widely used serological methods include ELISA, virus neutralization test (VNT), immunofluorescence assay (IFA), hemagglutination inhibition (HI) test, and latex agglutination test (LAT). These methods are indispensable in both field and laboratory settings, particularly in resource-limited areas, where rapid diagnosis is essential for disease control and eradication strategies.

Although serological tests, such as ELISA and VNT, are affordable and widely available, their use in large-scale monitoring requires consideration of cost and accessibility for farmers in developing regions. Therefore, ensuring low-cost production of commercial kits and establishing training programs for veterinary staff are crucial for the sustainability of field diagnostics.

## ELISA

ELISA remains the primary method for large-scale surveillance of porcine reproductive and respiratory syndrome (PRRS), pseudorabies (Aujeszky’s disease), and classical swine fever (CSF). Commercial kits are available to detect antibodies (indirect ELISA) or viral antigens (sandwich ELISA) in serum, oral fluid, or tissue homogenates.

A study comparing commercial ELISA kits for PRRSV demonstrated high sensitivity (>95%) and specificity when qPCR-confirmed samples were tested [11]. Additionally, oral fluid ELISA assays have gained popularity due to their ease of collection and reduced stress on animals.

The sensitivity and specificity of ELISA tests vary depending on the antigen used, the assay type (indirect, sandwich, or competitive), and the stage of infection. For example, an indirect ELISA for detecting antibodies to PCV2 has demonstrated a sensitivity of over 90% and specificity of approximately 95% in production settings [12–16]. A competitive ELISA for CSFV can detect antibodies even in the presence of maternal antibodies, which is particularly important when monitoring vaccination efficacy [17–20].

In recent years, innovations such as blocking ELISA and multiplex ELISA have significantly expanded the diagnostic capabilities of this platform. Multiplex ELISAs enable the simultaneous detection of antibodies to multiple viruses, saving time and resources while providing a more comprehensive picture of infection status in a single run [21–25].

## VNT

The VNT is considered the gold standard for detecting virus-specific neutralizing antibodies in the presence of multiple serotypes. The method involves mixing serum with live virus and adding the mixture to sensitive cell cultures; the absence of a cytopathic effect indicates the presence of neutralizing antibodies.

VNT is typically used as a confirmatory test following ELISA based serological screening. Although labor-intensive and time-consuming, it offers high specificity, allowing differentiation between vaccine strains and wild-type viruses in diseases such as CSF and PRRS [26].

VNT correlates strongly with protective immunity, particularly in diagnosing PRRSV, CSFV, and PRV. For example, a VNT for CSFV demonstrated 100% specificity and 95.6% sensitivity [27]. Despite requiring biosafety level 2 or 3 facilities, VNT remains indispensable for verifying ELISA results and monitoring vaccine efficacy [28–32].

In the case of PRRSV, VNT is performed using the Monkey African green kidney–derived cell line, clone 145 (MARC-145), or Porcine Alveolar Macrophages cells to determine the serum neutralizing activity against different viral strains. This is particularly relevant in regions where multiple PRRSV genotypes co-circulate, as continuous evaluation of vaccine effectiveness is required [33–37].

### HI test

The HI test is widely applied in diagnosing and monitoring SIV infections. This method is based on the ability of serum antibodies to inhibit viral hemagglutinin-induced red blood cell agglutination.

Despite the development of molecular diagnostic tools, the HI test remains a primary method in epidemiological studies and for assessing vaccine effectiveness in SIV surveillance [38–42]. It is relatively simple and cost-effective but requires strict standardization of parameters such as red blood cell concentration, viral titers, and antigen matching with circulating strains to ensure reproducible results.

### LAT

The LAT was developed as a rapid field serological method for detecting antibodies or antigens. It is easy to perform, does not require specialized equipment, and provides results within a few minutes.

However, LAT has lower sensitivity and specificity compared with ELISA and VNT, which limits its use primarily to preliminary screening rather than definitive diagnosis [43–45]. Despite these limitations, LAT remains a valuable tool in field conditions where rapid decision-making is essential, and laboratory resources are limited.

### Transition toward molecular diagnostics

Following advances in serological assays, molecular diagnostic technologies have rapidly evolved into more precise, high-throughput tools for pathogen detection. Methods such as qPCR, digital PCR (dPCR), and NGS now complement serological tests by enabling early detection, accurate quantification, and molecular characterization of viral pathogens, thereby enhancing overall diagnostic accuracy and surveillance efficiency.

## MOLECULAR DIAGNOSTIC TECHNOLOGIES FOR VIRAL INFECTIONS IN PIGS

Molecular technologies have become the “gold standard” for detecting viral nucleic acids due to their high sensitivity and specificity. The most widely used method in veterinary virology remains the PCR and its numerous modifications.

### Sample preparation and nucleic acid extraction

Efficient and contaminant-free extraction of nucleic acids is critical for obtaining reliable molecular diagnostic results. The most common methods use silica gel columns and magnetic beads to ensure high-quality DNA/RNA extraction. Recent innovations include mobile extraction devices integrated with portable PCR platforms, allowing on-site molecular testing for field applications [46].

### PCR and qPCR

Classical PCR amplifies specific regions of viral DNA or RNA, including those of PRRSV, PCV2, and CSFV. Real-time PCR (qPCR) complements this approach by enabling quantitative assessment while minimizing contamination risks through its closed-tube system.

This technology is widely used for routine monitoring and epizootic investigations due to its high speed, precision, and reproducibility. For example, in PRRSV diagnosis, qPCR allows reliable detection of the virus even at low viral loads in serum and tissue samples [47]. Modern multiplex qPCR assays can simultaneously detect and differentiate multiple pathogens, such as PCV2 and PCV3, in a single reaction [48].

Further advancements have been achieved with dPCR, which provides absolute quantification without the need for standard curves and demonstrates greater resistance to inhibitory substances. However, molecular diagnostics such as qPCR and NGS remain relatively expensive and technically demanding, limiting adoption in small or resource-limited farms. The development of portable PCR instruments and low-cost reagents could help bridge this gap between high-end laboratory testing and field-based diagnostics.

### dPCR and its quantitative applications

dPCR is a modern refinement of PCR that enables precise quantification of DNA or RNA copies without the need for standard calibration curves. The DNA sample is divided into thousands or millions of microreactions (droplets, wells, or nanoparticles). Each microreaction contains either one or no copies of the target sequence. After amplification, reactions are classified as positive (amplified) or negative (no amplification), and the absolute number of DNA copies in the original sample is calculated using Poisson’s law.

Types of systems include droplet dPCR (ddPCR), which partitions reactions into droplets (e.g., Bio-Rad QX200), and chip-based dPCR, which divides reactions into microwells on a chip (e.g., QuantStudio Absolute Q, Stilla Naica).

The main features of dPCR include high sensitivity and specificity, absolute quantification, robust reproducibility, and resistance to PCR inhibitors, giving it a significant advantage when working with viral nucleic acids [49].

Applications of dPCR in vaccine evaluation include:

1. Monitoring viral load post-vaccination (e.g., live attenuated vaccine trials or challenge studies)
2. Quantifying viral RNA/DNA or plasmid copy numbers in blood, tissues, or cells
3. Assessing inactivation efficiency in vaccine production (e.g., residual viral activity)
4. Measuring immune gene expression in vaccinated animals
5. Comparing immune responses across different vaccine doses or schedules [50, 51].

### Isothermal amplification methods (LAMP and RPA)

LAMP and RPA have gained prominence for field applications due to their simplicity, rapidity, and minimal equipment requirements. Both techniques operate at constant temperatures (60°C–65°C), eliminating the need for a thermal cycler.

Field studies in sub-Saharan Africa demonstrated that LAMP achieved >90% concordance with qPCR in detecting ASFV under field conditions [52]. Similarly, RPA has been successfully applied for diagnosing CSFV and SIV in resource-limited settings, delivering results in <20 min [53].

These isothermal methods have transformed field-based surveillance, enabling rapid, point-of-care diagnostics essential for early outbreak control.

### NGS and metagenomics

NGS has revolutionized swine viral diagnostics by enabling high-throughput, parallel detection of both known and novel pathogens. Unlike targeted PCR-based methods, NGS provides a non-targeted approach that simultaneously detects multiple viruses, identifies coinfections, and tracks genomic mutations.

Whole-genome sequencing using platforms such as Illumina, Oxford Nanopore Technologies (ONT), and PacBio has become integral to molecular epidemiology and phylogenetic studies of PRRSV, PCV2, and ASFV. For instance, the Illumina MiSeq platform (Illumina, CA, USA) has been used to analyze whole genomes of PRRSV strains in East Asia, revealing recombinant variants with altered virulence [53]. The Illumina MiSeq is an NGS system that uses sequencing-by-synthesis on flow cells with pre-packaged reagents, optical detection, and automated fluidics to produce high-quality genomic data. ONT’s portable MinION (Oxford Nanopore Technologies plc., UK) and PromethION (Oxford Nanopore Technologies plc., UK) devices have demonstrated the feasibility of mobile sequencing, offering rapid genomic data for ASFV and Avian Adenovirus detection [54]. ONT’s MinION and PromethION are nanopore-based sequencing devices that use flow cells with embedded nanopores and proprietary reagents to generate real-time genomic data. MinION is portable for field use, while PromethION offers high-throughput sequencing.

Furthermore, high-fidelity sequencing has proven effective in analyzing complex genomic structures and revealing quasispecies diversity in PRRSV and PCV2 coinfections [55].

Metagenomics, which sequences all nucleic acids within a sample, enables the identification of previously uncharacterized viruses, such as Swine Acute Diarrhea Syndrome Coronavirus, a growing concern for both pig health and public health [56].

Although NGS and metagenomics were once costly, they are now becoming increasingly accessible due to affordable sequencing chemistries and open-source bioinformatics tools, such as Kraken2, MetaPhlAn3, MEGAHIT, and SPAdes. Cloud-based platforms (e.g., BaseSpace, Galaxy) now allow sophisticated data processing even in resource-limited laboratories.

A typical NGS workflow includes nucleic acid extraction, library preparation, sequencing, and bioinformatic analysis. Tools such as Kraken2 and MetaPhlAn3 facilitate rapid taxonomic classification of reads and yield accurate insights into microbial community composition. These technologies are already in use in national surveillance programs for transboundary diseases like ASF [57].

Despite their advantages, NGS and metagenomics face challenges, including the need for skilled personnel and advanced computational infrastructure, as well as the risk of false-positive results from contamination or misclassification. Nevertheless, ongoing protocol standardization and cloud-based data systems are reducing these barriers, making NGS increasingly practical for veterinary diagnostics worldwide.

### Molecular targets in diagnostic assays

Molecular diagnostic methods have enabled the detection of major swine viral diseases with high accuracy and sensitivity. Techniques such as qPCR, RT-PCR, and NGS have proven effective in pathogen identification and genetic characterization.

To ensure diagnostic consistency, highly conserved genomic regions are typically used as targets, including Open Reading Frame 5 (ORF5) in PRRSV, Cap in PCV2, p72 in ASFV, and E2 in CSFV. These genes serve as reliable molecular markers across diagnostic platforms, PCR, qPCR, NGS, serological assays, and emerging CRISPR-based detection systems. The key viral gene targets used in molecular diagnostics of swine viruses are summarized in Table 1.

**Table 1:** Key genetic targets and their diagnostic applications in swine viral infections.

Virus	Key diagnostic genes	Function	Diagnostic tools
PRRSV	*ORF5, nsp2*	Envelope glycoprotein and replication enzyme	qPCR, ELISA, and WGS
PCV2	*Cap (ORF2), Rep (ORF1)*	Capsid protein, an initiator of replication	qPCR, ELISA, and NGS
ASFV	*p72, p30, CD2v*	Structural protein and hemadsorption factor	PCR, LAMP, and CRISPR/Cas
CSFV	*E2, NS5B*	Surface glycoprotein and RNA polymerase	RT-qPCR, ELISA, and VNT
PEDV	*N, S*	Nucleocapsid, spike protein	qPCR, LAMP, and POCT
SVA	*VP1, 3Dpol*	Capsid and polymerase genes	RT-PCR, ELISA, and NGS
TTSuV	*ORF1, VP1*	Capsid and replication-associated proteins	qPCR, metagenomics

PEDV = Porcine epidemic diarrhea virus, POCT = Point-of-care testing, WGS = Whole-genome sequencing, PRRSV = Porcine reproductive and respiratory syndrome virus, PCV = Porcine circovirus type 2, ASFV = African swine fever virus, CSFV = Classical swine fever virus, qPCR = Quantitative polymerase chain reaction, ELISA = Enzyme-linked immunosorbent assay, NGS = Next-generation sequencing, LAMP = Loop-mediated isothermal amplification, CRISPR = Clustered regularly interspaced short palindromic repeats, RT-qPCR = Reverse-transcription quantitative polymerase chain reaction, VNT = Virus neutralization test, SVA = Senecavirus A, TTSuV = Torque Teno Sus Virus, ORF = Open Reading Frame, VP = Viral Protein.

## EMERGING DIAGNOSTIC TECHNOLOGIES

Modern diagnostic science has entered a new phase of rapid innovation, integrating molecular precision with digital intelligence. Emerging tools such as CRISPR/Cas-based systems, lab-on-a-chip (LOC) platforms, POCT devices, and nanotechnology-enabled biosensors are revolutionizing how swine viral infections are detected, monitored, and managed in both laboratory and field environments.

### CRISPR/Cas-based diagnostics

Recent advances in genome-editing technologies have led to the development of CRISPR/Cas-based diagnostic systems, particularly the Specific High-sensitivity Enzymatic Reporter unLOCKing and DNA Endonuclease-Targeted CRISPR Trans Reporter platforms. These methods utilize Cas13 or Cas12 enzymes to recognize specific nucleic acid sequences, followed by cleavage of reporter molecules that generate measurable signals.

CRISPR-based diagnostics have been successfully tested for the rapid detection of ASFV and PRRSV in swine virology, demonstrating high sensitivity comparable to PCR and achieving a turnaround time of <1 h [58]. The introduction of lyophilized CRISPR reagents has further improved their field applicability, allowing on-site testing without the need for cold-chain storage or complex instrumentation.

### Microfluidic platforms and LOC systems

In recent years, there has been growing scientific interest in microfluidic platforms and LOC systems. These miniature devices are designed to perform one or more laboratory analyses on a single microchip. Their applications extend across numerous domains, including disease diagnosis, clinical biochemistry (e.g., glucose, international normalized ratio, blood gases), nucleic acid detection, cell biology research (organ-on-a-chip), biomedical tissue engineering, environmental monitoring, and food safety [59, 60].

LOC systems enable multiplex testing using minimal sample volumes, making them particularly useful in resource-limited settings. Advantages of this technology include low reagent consumption, miniaturization, rapid analysis, portability, automation, and mass production potential [61, 62].

However, the technology still faces several challenges. The disadvantages of LOC systems include complex design and manufacturing, limited versatility, fluidic control issues, calibration and standardization difficulties, high equipment costs, and sometimes restricted sensitivity [63]. Despite these limitations, the continued evolution of LOC devices promises to make high-throughput diagnostics accessible even in remote environments.

### Rapid tests and POCT

POCT devices, including lateral flow immunoassays and portable PCR systems, allow veterinarians to detect viral infections directly on farms, significantly reducing outbreak response times.

Commercial POCT kits are currently available for detecting ASFV, PCV2, and PEDV. Comparative studies have shown that portable isothermal devices employing LAMP or RPA achieved over 90% concordance with laboratory-based qPCR assays in field conditions [64].

Some portable systems integrate sample preparation, amplification, and result visualization into a single device. These instruments can connect to smartphones or Bluetooth-enabled printers, making them ideal for use in remote or low-resource settings.

POCT technologies, especially those based on LAMP and RPA, are recognized for their affordability, rapid turnaround, and minimal equipment requirements. They offer a strong cost–benefit ratio, as early detection of infections can prevent large-scale economic losses associated with culling, movement restrictions, and trade disruptions.

### Biosensors and nanotechnology-based devices

In recent years, biosensors and digital surveillance systems have transformed veterinary diagnostics by enabling real-time, on-site detection of viral agents directly at the production level. These tools represent a new generation of molecular–digital integration, bridging laboratory precision with field practicality.

Nanotechnology-based biosensors incorporating gold nanoparticles, graphene, and quantum dots are increasingly being adopted for swine disease diagnostics. These platforms detect viral proteins or nucleic acids in real-time with high sensitivity and specificity.

Moreover, wearable biosensor platforms that continuously monitor physiological parameters, such as body temperature, respiration rate, and metabolic biomarkers, are being developed for livestock disease surveillance. Such devices facilitate early detection of subclinical viral infections, enhancing proactive herd management.

When integrated with cloud-based databases, Internet of Things (IoT) networks, and AI-driven analytics, biosensors enable real-time tracking of disease distribution and support predictive modeling for outbreak management. These technologies are increasingly being incorporated into Precision Livestock Farming systems, where digital monitoring enhances decision-making and biosecurity at the herd-level.

For instance, an electrochemical graphene-based biosensor successfully detected the PCV2 capsid protein in serum samples within 10 min [53]. When coupled with cloud-based data collection platforms, such biosensors facilitate real-time mapping of disease spread and contribute to early warning systems that improve epidemic response.

Despite their transformative potential, sequencing-based and digital systems still require substantial computational resources, skilled personnel, and robust quality control frameworks. In this context, AI and automated digital systems are increasingly employed to streamline data interpretation, optimize diagnostic workflows, and enhance real-time outbreak response.

## DIGITALIZATION AND AI IN DIAGNOSTICS

The increasing integration of digital technologies, AI, and molecular diagnostics is transforming how swine viral diseases are monitored, analyzed, and controlled. Integration of AI, cloud computing, and real-time genomic data enables faster, more accurate decision-making, thereby strengthening early warning systems and global biosurveillance under the One Health framework.

### AI and machine learning (ML) in diagnostic interpretation

The incorporation of AI and ML has recently transformed swine disease diagnostics by enabling automated data interpretation, early outbreak prediction, and real-time epidemiological modeling. AI-powered algorithms can analyze imaging data from IFAs, microarrays, or histopathological slides with remarkable precision, reducing human bias and increasing diagnostic throughput [65]. ML models trained on qPCR and sequencing data facilitate the early detection of emerging viral strains and the prediction of mutation trends [66]. For example, deep learning frameworks integrated with genomic datasets can differentiate between vaccine-derived and field strains of PRRSV, thereby optimizing vaccination strategies and biosurveillance efficiency [67].

### AI and IoT integration for real-time monitoring

The fusion of AI with IoT technologies has enabled the development of intelligent diagnostic platforms. Automated sample collection, cloud-based data storage, and real-time dashboards now allow veterinarians to monitor disease dynamics at both the herd and regional levels.

GISs and predictive algorithms further enhance outbreak mapping, risk modeling, and intervention timing, effectively reducing the likelihood of pathogen transmission between farms [68]. These integrated systems form the foundation of smart veterinary surveillance networks, facilitating data-driven disease control decisions.

### Global GS and data-sharing networks

The growing interconnection between diagnostic systems and GS networks has transformed the global monitoring of swine viral diseases. International data-sharing initiatives such as the Global Initiative on Sharing All Influenza Data provide real-time access to genomic sequences of influenza A subtypes (H1N1 and H3N2), enabling early detection of reassortant strains with zoonotic potential [69]. Similarly, the FAO and WOAH coordinate regional genomic monitoring networks for ASFV, facilitating the rapid exchange of sequence data and molecular epidemiology results among national veterinary laboratories [70]. These cooperative systems enable harmonized phylogenetic tracking of transboundary viruses and improve outbreak containment efficiency.

### Visualization and analytical tools for viral evolution

Platforms such as Nextstrain and Pathogenwatch have enhanced visualization of viral evolution by integrating sequencing data from multiple sources into interactive dashboards [71, 72]. These visualization tools enable veterinary researchers to track ASFV and PRRSV lineages in real time, support genomic-informed decision-making, and aid in vaccine strain selection. Such interactive systems are instrumental in understanding virus transmission dynamics and predicting evolutionary trends at both local and global scales.

### Mobile and cloud-based surveillance applications

Emerging mobile surveillance applications, including EpiCollect5 (developed by the CGPS Team at the Oxford University Big Data Institute, UK), Open Data Kit (https://getodk.org/), and VaccineGuard (https://guardtime. com/vaccineguard), demonstrate how digital reporting and GPS-enabled data collection can streamline outbreak response, especially in low-resource environments [73]. EpiCollect5, Open Data Kit, and VaccineGuard are mobile data collection platforms. They enable GPS-enabled surveys, real-time reporting, and cloud-based data management, streamlining outbreak response, especially in low-resource settings.

These tools allow veterinary authorities to synchronize near-real-time diagnostic data from the field with centralized databases, thereby improving data accuracy, disease trend visualization, and predictive modeling. Integration of these platforms with laboratory and genomic databases enhances traceability and rapid response capacity during epidemics.

### One Health integration and the future of digital diagnostics

The integration of laboratory diagnostics with global GS networks is a cornerstone of the One Health approach, linking on-farm testing with international biosurveillance frameworks. Such digital connectivity is vital for strengthening preparedness against transboundary and zoonotic viral threats. AI-powered digital tools not only enhance diagnostic precision but also enable cross-sector data integration between veterinary and public health systems. The combination of AI-driven analytics with genomic and serological data represents a key advancement toward precision livestock health management, ensuring sustainable and resilient disease monitoring under the One Health paradigm [74]. The current diagnostic methods for swine viral diseases are summarized in Table 2 [75–87].

**Table 2:** Comparison of the performance, strengths, and limitations of molecular and serological diagnostic methods for swine viral infections.

Diagnostic method	Main target	Time to results	Field usability	Sensitivity	Reference
ELISA	Antibodies/Antigens	2–5 h	Moderate	High	[75]
VNT	Neutralizing antibodies	3–7 days	Low	Very High	[76]
HI test	Blocking virus-induced RBC agglutination	2–3 days	High	Moderate	[77]
The LAT	Viral or bacterial antigens (specific antibodies)	5–20 min	Very High	Moderate to high	[78]
PCR	Viral DNA/RNA	3–5 h	Low	Moderate	[79]
qPCR (multiplex)	Viral DNA/RNA	2-6 h	High	Very High/High	[80]
dPCR	Genomes	3-8 h	High	Very High	[81]
LAMP	Conserved viral genes	30–60 min	High	High	[82]
NGS	Viral genomes (known and unknown)	24–72 h	Low	High	[82]
CRISPR/Cas-based Diagnostics	Viral DNA/RNA	<1 h	High	Very High	[83]
Microfluidic Platforms and Lab-on-a-Chip	Viral nucleic acids (DNA/RNA) or proteins	15–60 min	High	Very High	[84]
POCT	Antigens/Genomes	<2 h	Very High	High	[85]
Biosensors	Proteins and Genomes	<30 min	High	Moderate to high	[86]
AI	Multisource diagnostic data (clinical, genomic, imaging, and epidemiological) for the automated detection and prediction of viral infections	Real-time	High	High	[87]

ELISA = Enzyme-linked immunosorbent assay, VNT = Virus neutralization test, HI = Hemagglutination inhibition, LAT = Latex agglutination test, PCR = Polymerase chain reaction, qPCR = Quantitative PCR, dPCR = Digital PCR; LAMP = Loop-mediated isothermal amplification, NGS = Next-generation sequencing, CRISPR = Clustered regularly interspaced short palindromic repeats, POCT = Point-of-care testing, BSL = Biosafety level, AI = Artificial intelligence, RBC = Red blood cell.

## RECENT ADVANCES AND FUTURE DIRECTIONS

The rapid evolution of molecular, serological, and digital diagnostic technologies has significantly enhanced the detection, monitoring, and management of porcine viral diseases. Comparative analyses from recent studies demonstrate that while qPCR remains the gold standard for routine diagnostics, emerging technologies such as dPCR, metagenomic sequencing, and CRISPR-based assays are redefining precision and expanding pathogen discovery capabilities.

### Progress in molecular diagnostic tools

Molecular assays continue to set the benchmark for speed, accuracy, and sensitivity in swine virology. Comparative findings have shown that qPCR targeting conserved genomic regions, such as *PRRSV ORF5* and *ASFV* genes, offers superior analytical sensitivity compared with conventional PCR [88]. Similarly, dPCR provides greater quantification accuracy in mixed infections of PRRSV and PCV2, allowing better differentiation between viral loads in co-infected samples [89].

These findings confirm that modern molecular platforms not only improve diagnostic precision but also enhance the capacity to monitor viral genetic diversity and evolution, supporting early warning systems for emerging variants.

### The continuing role of serological techniques

Although newer molecular methods dominate, traditional serological assays remain indispensable for herd-level surveillance and vaccine monitoring. Comparative studies by Shin *et al*. [14] and Cao *et al*. [18] demonstrated that ELISA remains cost-effective and suitable for mass screening, particularly using oral fluid samples.

While VNTs offer high specificity, their reliance on biosafety infrastructure limits field deployment. In contrast, CRISPR-based molecular assays targeting ASFV p72 and PCV2 Cap genes enable rapid strain differentiation between vaccine and field isolates [90]. However, as Wei *et al*. [64] noted, CRISPR diagnostics still require extensive field validation, particularly to address temperature sensitivity and reagent stability challenges in rural or mobile laboratories.

### Advances in NGS and metagenomics

Recent progress in NGS has expanded diagnostic horizons by enabling high-throughput, parallel detection of known and novel pathogens. Illumina MiSeq and Oxford Nanopore platforms have proven particularly effective for GS and mutation tracking, essential for tracing transboundary outbreaks

Despite their diagnostic power, NGS applications remain limited by high operational costs and the need for bioinformatics expertise. As Xu *et al*. [57] observed, the accuracy of metagenomic classification depends heavily on the completeness of reference databases, making misclassification a persistent concern.

To address these challenges, FAO and WOAH recommend standardization of bioinformatic workflows and international data-sharing frameworks, ensuring reproducibility and global accessibility of genomic insights.

### LOC and microfluidic innovation

LOC technology represents a major advance in diagnostic miniaturization and automation. By integrating sample preparation, amplification, and analysis into a single microplatform, LOC systems significantly improve analytical efficiency, accuracy, and reagent economy.

These systems are highly valued in medicine, biotechnology, and environmental diagnostics, offering rapid processing and on-site testing potential. However, challenges persist, including high equipment costs, complex standardization, and component integration difficulties. Despite these barriers, LOC technology holds immense potential for future scientific and applied veterinary diagnostics.

### Balancing cost, accessibility, and performance

From a practical perspective, achieving an optimal cost–efficiency balance remains central to diagnostic sustainability. While qPCR and LAMP assays are increasingly affordable and standardized, advanced systems such as NGS and CRISPR have yet to achieve comparable accessibility.

Huang et al. reported that LAMP-based Q-POC devices achieved >90% diagnostic accuracy for ASFV under field conditions [91], offering a promising alternative for outbreak response in resource-limited regions. Such on-site testing platforms align with the FAO Progressive Control Pathway for ASF, supporting rapid containment and prevention of disease spread.

### Strengths and limitations of dPCR

While dPCR and ddPCR offer unmatched quantification accuracy, they also face limitations. Sensitivity may decline due to small reaction volumes, and measurement ranges depend on platform capacity and fragment availability.

Moreover, dPCR does not differentiate between live and inactivated viruses and remains slower and costlier than qPCR [92]. Nonetheless, ddPCR is projected to become a powerful diagnostic platform for viral load quantification, single-nucleotide polymorphism detection, and virus–host interaction studies [93].

### AI and global harmonization

Integration of AI and digital analytics has become a key dimension of next-generation diagnostics. ML models trained on genomic datasets enable early prediction of outbreak dynamics, mutation emergence, and automated pathogen classification.

Silvia *et al*. [65] and Choi *et al*. [66] reported that AI-driven diagnostic algorithms significantly enhance interpretation accuracy, automate pathogen identification, and reinforce One Health surveillance strategies. However, policy harmonization remains a barrier, differences in national data standards, biosecurity regulations, and laboratory capacities hinder global interoperability.

Under the guidance of WOAH and FAO, international coordination is essential to establish uniform diagnostic criteria, data-sharing protocols, and biosurveillance standards that support equitable access and data security.

### Persistent challenges and knowledge gaps

Despite substantial progress, several knowledge gaps continue to affect diagnostic reliability:

- Genetic variability of PRRSV and ASFV complicates the design of universal primers and stable targets
- Coinfections (e.g., PRRSV–PCV2, PRRSV–PEDV) obscure clinical interpretation and reduce specificity
- Metagenomic analyses, while powerful, often struggle to distinguish between clinically relevant and incidental viral sequences.

Additionally, coinfections with secondary bacterial agents can mask viral signatures, decreasing assay sensitivity. Rapid viral evolution in PRRSV and SIV drives genetic drift, necessitating constant assay updates. Integrating GS with routine diagnostics is therefore vital to maintain test relevance and performance.

### Regulatory, ethical, and biosafety considerations

The future of veterinary diagnostics depends on regulatory validation, ethical data governance, and preparedness for emerging pathogens. Platforms such as CRISPR, NGS, and POCT require standardized validation and licensing under WOAH, European Food Safety Authority (EFSA), and *United States Department of Agriculture – Animal and Plant Health Inspection Service* (USDAAPHIS) frameworks to ensure accuracy and biosafety.

Ethical concerns focus on secure genomic data management and biosafety controls for portable sequencing devices. The increasing awareness of “Disease X”, unknown future pathogens, underscores the importance of metagenomics and AI-assisted surveillance for rapid identification of novel viral threats.

### Global disparities and diagnostic capacity

Modern diagnostics serve as strategic tools for biosafety, trade, and food security. However, implementation disparities remain between high-income and low-resource systems. Access to CRISPR/Cas, NGS, and dPCR is limited in developing regions due to cost, infrastructure, and training constraints.

Encouragingly, low-cost POCT and LAMP assays are increasingly bridging this gap, supporting field-based surveillance in rural areas. Alignment with WOAH and FAO biosafety standards enhances international trade transparency and strengthens transboundary disease reporting systems.

### Future outlook and One Health integration

Reliable diagnostic capacity underpins global food security and the One Health paradigm, connecting animal health monitoring with human health preparedness and environmental sustainability.

Regional trends illustrate diagnostic diversity:

1)Asia: Widespread use of LAMP and rapid PCR for ASFV outbreaks
2)Europe: Established qPCR–ELISA networks for PRRSV and PCV2 surveillance
3)North America: Increasing use of NGS and dPCR for PEDV and SIV monitoring.

Reducing diagnostic disparities requires harmonized international standards, robust data-sharing frameworks, and capacity building in developing regions.

**Figure 1:**
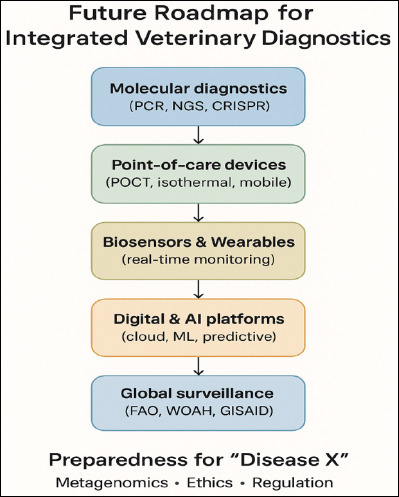
Future roadmap for integrated veterinary diagnostics, showing progressive integration of molecular diagnostics, point-of-care devices, biosensors, artificial intelligence, and global surveillance systems to enhance preparedness for emerging diseases (“Disease X”).

The future of veterinary diagnostics lies in the convergence of molecular tools, intelligent sensing, and AI-driven analytics. As illustrated in Figure 1, this roadmap envisions the integration of emerging technologies into global surveillance systems designed for preparedness against novel and re-emerging pathogens, including “Disease X.”

In conclusion, contemporary diagnostic advancements highlight the integration of precision molecular assays, rapid point-of-care technologies, and AI-driven analytical systems. The effective and affordable implementation of these tools, particularly within the frameworks of global trade, biosecurity, and One Health, demands not only continued technological innovation but also policy harmonization across international veterinary networks. Strengthening global collaboration under the guidance of the FAO and WOAH will be essential to ensure that emerging diagnostic innovations are translated into practical, field-ready solutions, ultimately enhancing the early detection, control, and prevention of porcine viral diseases worldwide.

## CONCLUSION

This review highlights significant progress in the diagnostic landscape for porcine viral diseases, driven by the integration of advanced molecular techniques, innovative serological assays, and digital surveillance systems. The comparative analysis of recent studies demonstrates that qPCR continues to serve as the gold standard for routine laboratory detection due to its high sensitivity and reproducibility, while dPCR, LAMP, and NGS-based approaches have expanded diagnostic precision, enabling rapid pathogen quantification, co-infection profiling, and GS. The emergence of CRISPR/Cas-based assays, LOC systems, and AI-driven analytics further represents a transformative shift toward rapid, field-deployable, and data-integrated veterinary diagnostics.

From a practical standpoint, these technologies offer substantial benefits for early outbreak detection, disease mapping, and biosecurity decision-making. The use of portable and low-cost POCT devices, especially LAMP and RPA platforms, enhances accessibility in low-resource settings and supports real-time disease control aligned with FAO’s Progressive Control Pathway for transboundary animal diseases. Moreover, the integration of AI, IoT, and GIS frameworks provides a predictive capability, allowing for dynamic risk assessment and improved response coordination under the One Health paradigm.

The strengths of modern diagnostic systems lie in their sensitivity, specificity, speed, and adaptability. They allow for multiplex detection, real-time genomic monitoring, and the identification of novel pathogens through metagenomics. However, limitations persist, including high operational costs, the need for trained personnel, a lack of standardization in bioinformatic pipelines, and limited interoperability between national and regional diagnostic networks. The diagnostic performance of newer tools, particularly CRISPR-based and isothermal assays, still requires extensive field validation under variable environmental and epidemiological conditions.

Looking to the future, research should focus on improving assay portability, reducing reagent costs, and establishing global data-sharing frameworks to harmonize GS. Strengthening international collaboration under the guidance of the FAO and WOAH will be critical to ensuring quality assurance, standardized validation, and equitable access to diagnostic technologies worldwide. The continued convergence of molecular precision, digital intelligence, and ethical governance will define the next generation of diagnostic preparedness.

In summary, modern diagnostic tools are no longer merely laboratory instruments; they are strategic components of global animal health security, linking science, technology, and policy. By ensuring cost-effective implementation, cross-sector collaboration, and continuous innovation, the veterinary community can achieve earlier detection, stronger control, and sustainable prevention of porcine viral diseases, ultimately safeguarding both livestock productivity and One Health resilience.

## AUTHORS’ CONTRIBUTIONS

KN: Conceived and designed the study, developed the research concept, performed the literature search, and drafted the original manuscript. AR: Provided supervision, project administration. DA: Carried out statistical analysis and assisted in the preparation of the results. DS: Collected and curated data and prepared tables. VS: Conducted the methodological analysis and contributed to data interpretation. All authors have read and approved the final version of the manuscript.
